# Surveillance for Coccidioidomycosis, Histoplasmosis, and Blastomycosis — United States, 2019

**DOI:** 10.15585/mmwr.ss7107a1

**Published:** 2022-08-19

**Authors:** Dallas J. Smith, Samantha L. Williams, Endemic Mycoses State Partners Group, Kaitlin M. Benedict, Brendan R. Jackson, Mitsuru Toda, Endemic Mycoses State Partners Group, Guillermo Adame, Laura Rothfeldt, Gail Sondermeyer Cooksey, Kristen Rios, Connie Austin, Mary-Elizabeth Steppig, Sheri Tubach, Natalie Christophe, Kristy Lunquest, Rebecca Reik, Malia Ireland, Danny Power, Deanna Bridges, Laura Cronquist, Katie Cibulskas, Paul Cieslak, Kimberly Warren, Michael Gosciminski, Dustin Ortbahn, BreAnne Osborn, Hanna Oltean, Suzanne Gibbons-Burgener

**Affiliations:** ^1^Epidemic Intelligence Service; ^2^Mycotic Diseases Branch, Division of Foodborne, Waterborne, and Environmental Diseases, CDC; Arizona Department of Health Services; Arkansas Department of Health; California Department of Public Health Infectious Diseases Branch; Delaware Division of Public Health; Illinois Department of Public Health; Indiana Department of Health; Kansas Department of Health and Environment; Louisiana Department of Health; Maryland Department of Health; Michigan Department of Health and Human Services; Minnesota Department of Health; Montana Department of Public Health and Human Services; New Hampshire Division of Public Health Services; North Dakota Department of Health; Ohio Department of Health; Oregon Health Authority Public Health Division; Pennsylvania Department of Health; Rhode Island Department of Health; South Dakota Department of Health; Utah Department of Health; Washington State Department of Health; Wisconsin Department of Health Services.

## Abstract

**Problem/Condition:**

Coccidioidomycosis, histoplasmosis, and blastomycosis are underdiagnosed fungal diseases that often mimic bacterial or viral pneumonia and can cause disseminated disease and death. These diseases are caused by inhalation of fungal spores that have distinct geographic niches in the environment (e.g., soil or dust), and distribution is highly susceptible to climate changes such as expanding arid regions for coccidioidomycosis, the northward expansion of histoplasmosis, and areas like New York reporting cases of blastomycosis previously thought to be nonendemic. The national incidence of coccidioidomycosis, histoplasmosis, and blastomycosis is poorly characterized.

**Reporting Period:**

2019.

**Description of System:**

The National Notifiable Diseases Surveillance System (NNDSS) tracks cases of coccidioidomycosis, a nationally notifiable condition reported to CDC by 26 states and the District of Columbia. Neither histoplasmosis nor blastomycosis is a nationally notifiable condition; however, histoplasmosis is voluntarily reported in 13 states and blastomycosis in five states. Health departments classify cases based on the definitions established by the Council of State and Territorial Epidemiologists.

**Results:**

In 2019, a total of 20,061 confirmed coccidioidomycosis, 1,124 confirmed and probable histoplasmosis, and 240 confirmed and probable blastomycosis cases were reported to CDC. Arizona and California reported 97% of coccidioidomycosis cases, and Minnesota and Wisconsin reported 75% of blastomycosis cases. Illinois reported the greatest percentage (26%) of histoplasmosis cases. All three diseases were more common among males, and the proportion for blastomycosis (70%) was substantially higher than for histoplasmosis (56%) or coccidioidomycosis (52%). Coccidioidomycosis incidence was approximately four times higher for non-Hispanic American Indian or Alaska Native (AI/AN) persons (17.3 per 100,000 population) and almost three times higher for Hispanic or Latino persons (11.2) compared with non-Hispanic White (White) persons (4.1). Histoplasmosis incidence was similar across racial and ethnic categories (range: 0.9–1.3). Blastomycosis incidence was approximately six times as high among AI/AN persons (4.5) and approximately twice as high among non-Hispanic Asian and Native Hawaiian or other Pacific Islander persons (1.6) compared with White persons (0.7). More than one half of histoplasmosis (54%) and blastomycosis (65%) patients were hospitalized, and 5% of histoplasmosis and 9% of blastomycosis patients died. States in which coccidioidomycosis is not known to be endemic had more cases in spring (March, April, and May) than during other seasons, whereas the number of cases peaked slightly in autumn (September, October, and November) for histoplasmosis and in winter (December, January, and February) for blastomycosis.

**Interpretation:**

Coccidioidomycosis, histoplasmosis, and blastomycosis are diseases occurring in geographical niches within the United States. These diseases cause substantial illness, with approximately 20,000 coccidioidomycosis cases reported in 2019. Although substantially fewer histoplasmosis and blastomycosis cases were reported, surveillance was much more limited and underdiagnosis was likely, as evidenced by high hospitalization and death rates. This suggests that persons with milder symptoms might not seek medical evaluation and the symptoms self-resolve or the illnesses are misdiagnosed as other, more common respiratory diseases.

**Public Health Action:**

Improved surveillance is necessary to better characterize coccidioidomycosis severity and to improve detection of histoplasmosis and blastomycosis. These findings might guide improvements in testing practices that enable timely diagnosis and treatment of fungal diseases. Clinicians and health care professionals should consider coccidioidomycosis, histoplasmosis, and blastomycosis in patients with community-acquired pneumonia or other acute infections of the lower respiratory tract who live in or have traveled to areas where the causative fungi are known to be present in the environment. Culturally appropriate tailored educational messages might help improve diagnosis and treatment. Public health response to these three diseases is hindered because information gathered from states’ routine surveillance does not include data on populations at risk and sources of exposure. Broader surveillance that includes expansion to other states, and more detail about potential exposures and relevant host factors can describe epidemiologic trends, populations at risk, and disease prevention strategies.

## Introduction

Coccidioidomycosis, histoplasmosis, and blastomycosis are fungal diseases that can infect anyone, regardless of immune status, and can progress to life-threatening severe pulmonary or disseminated disease (*1*–*3*). Infections are typically acquired by inhalation of fungal spores from the environment, often in soil or dust. Climate change has the potential to expand the geographic range of coccidioidomycosis, histoplasmosis, and blastomycosis in the United States and globally (*4*–*7*). Coccidioidomycosis, also known as Valley fever and caused by *Coccidioides immitis* and *Coccidioides posadasii*, is most commonly acquired in the southwestern United States, although it is also acquired as far north as Washington (Supplementary Figure 1, https://stacks.cdc.gov/view/cdc/118602). Histoplasmosis, caused by *Histoplasma capsulatum,* is acquired primarily in central and eastern states, although the disease likely also occurs to lesser extents across much of the country (Supplementary Figure 2, https://stacks.cdc.gov/view/cdc/118603). Blastomycosis, caused primarily by *Blastomyces dermatitidis* and *Blastomyces gilchristii,* is found in midwestern, south-central, and southeastern states (Supplementary Figure 3, https://stacks.cdc.gov/view/cdc/118604) (*8*–*11*). The recently described *Blastomyces helicus* has been found to cause illness in the western United States but remains poorly understood (*12*). National surveillance of coccidioidomycosis, histoplasmosis, and blastomycosis can identify sources of disease and exposures among persons living in areas where the diseases are not known to be endemic. More information about the estimated areas with the fungi that cause coccidioidomycosis, histoplasmosis, and blastomycosis is available at https://www.cdc.gov/fungal/pdf/more-information-about-fungal-maps-508.pdf.

Coccidioidomycosis, histoplasmosis, and blastomycosis are frequently misdiagnosed as community-acquired pneumonia or other acute lower respiratory tract infections. Misdiagnoses can lead to inappropriate therapy with antibacterial medications and delayed antifungal treatment (*8*,*13*,*14*). Antifungal treatment is recommended based on clinical manifestation and severity of disease (*2*,*15*,*16*). Persons with weakened immune systems are at higher risk for severe disease; epidemiologic studies suggest that Black and Pacific Islander persons are also at higher risk for severe disease, and further study is needed to determine the reasons (*17*–*27*). Diagnosis of these diseases is complex because of variable clinical presentation, nonspecific chest imaging findings, and resource-intensive diagnostic tests (*28*,*29*).

Previously, each of these diseases has been described separately (*8*,*30*,*31*). This report summarizes 2019 U.S. surveillance data on coccidioidomycosis, histoplasmosis, and blastomycosis to examine and compare geographic distribution, populations at risk, and seasonality. The findings in this report can be used to raise awareness among public health professionals, health care providers, policymakers, and the public to improve timely diagnosis and treatment.

## Methods

### Data Source and Collection 

CDC uses the National Notifiable Diseases Surveillance System (NNDSS) to track coccidioidomycosis and histoplasmosis from states where the diseases are reportable by mandate and from those where cases are reported voluntarily. Reportable fungal diseases are designated by the state or jurisdiction and require health care professionals and laboratories to notify public health departments of cases. Nationally notifiable diseases such as coccidioidomycosis involve states or jurisdictions voluntarily submitting case data to CDC through NNDSS. Coccidioidomycosis surveillance data were submitted by 26 states (Alabama, Arizona, Arkansas, California, Delaware, Indiana, Kansas, Louisiana, Maryland, Michigan, Minnesota, Missouri, Montana, Nebraska, Nevada, New Hampshire, New Mexico, North Dakota, Ohio, Oregon, Rhode Island, South Dakota, Utah, Washington, Wisconsin, and Wyoming) and the District of Columbia, where it is reportable; in Washington, coccidioidomycosis is reportable as a rare disease of public health significance. All states and jurisdictions where coccidioidomycosis is reportable routinely submit data to NNDSS. Because of the COVID-19 pandemic, the 2019 coccidioidomycosis NNDSS data for California were incomplete; therefore, California submitted state surveillance data directly to CDC’s Mycotic Diseases Branch. Histoplasmosis and blastomycosis are not nationally notifiable, and data for these diseases are not submitted to NNDSS by all states where they are reportable. Histoplasmosis information included cases in NNDSS or data directly submitted to CDC from 13 state health departments (Arkansas, Delaware, Illinois, Indiana, Kansas, Kentucky, Louisiana, Michigan, Minnesota, Nebraska, Pennsylvania, Rhode Island, and Wisconsin); in Rhode Island, histoplasmosis is reportable as a rare or unusual condition. Only Delaware, Illinois, Kentucky, Louisiana, Michigan, Minnesota, Pennsylvania, Rhode Island, and Wisconsin submit data to NNDSS. Blastomycosis surveillance data were directly submitted to CDC from five state health departments (Arkansas, Louisiana, Michigan, Minnesota, and Wisconsin). NNDSS included case-level data, whereas the direct submissions to CDC contained aggregate data. Certain state and local health departments collected clinical and exposure information during their investigations and patient interviews; however, those types of data are not captured in NNDSS for coccidioidomycosis, histoplasmosis, or blastomycosis. States that submitted histoplasmosis and blastomycosis data directly to CDC also provided information on hospitalizations and deaths.

### Surveillance Case Definitions

The Council of State and Territorial Epidemiologists (CSTE) established national case definitions for coccidioidomycosis in 2011, histoplasmosis in 2017, and blastomycosis in 2019 (*32*–*34*). The 2011 CSTE coccidioidomycosis definition includes both laboratory and clinical criteria; cases can be classified as confirmed (*32*). Confirmed cases meet the clinical criteria and are laboratory confirmed. The laboratory criteria include culture, histopathologic, and molecular evidence of *Coccidioides* spp. or a positive serologic test for *Coccidioides* antibodies. Clinical criteria include symptoms resembling influenza or pneumonia-like illnesses, or the infection can be asymptomatic. In 2019, California and Arizona case data were based on laboratory-only reporting.

The 2017 CSTE histoplasmosis definition includes both laboratory and clinical criteria; cases can be classified as confirmed or probable (*33*). Confirmed cases are clinically compatible with histoplasmosis and meet confirmatory laboratory criteria. Probable cases are 1) clinically compatible and meet nonconfirmatory laboratory criteria, 2) meet confirmatory laboratory criteria but not clinical criteria, or 3) clinically compatible and do not meet laboratory criteria but are epidemiologically linked to a confirmed case. Clinical criteria include symptoms indicative of histoplasmosis, abnormal chest imaging findings, or evidence of disseminated disease. Confirmatory laboratory criteria include culture, histopathology, molecular detection, detection of H band by immunodiffusion antibody test or detection of M band after a previous lack of M band, or a greater than fourfold rise in antibody titer. Nonconfirmatory laboratory criteria include cytopathology or a single positive antibody or antigen enzyme immunoassay (EIA) test.

The 2019 CSTE blastomycosis definition includes both laboratory and clinical criteria; cases can be classified as confirmed or probable (*34*). Confirmed cases are clinically compatible with blastomycosis and meet confirmatory laboratory criteria. Probable cases are 1) clinically compatible and meet presumptive laboratory criteria, 2) clinically compatible and do not meet laboratory criteria but are epidemiologically linked to a confirmed case, or 3) laboratory confirmed but no clinical information is available. Clinical compatibility requires relevant symptoms, abnormal chest imaging findings, or evidence of disseminated disease. Confirmatory laboratory criteria include identification of *Blastomyces* by culture, histopathology, cytopathology, or molecular detection. Presumptive laboratory criteria include detection of *Blastomyces* antigen at or above the minimum level of quantification in serum, urine, or other body fluid by EIA test or detection in serum of antibodies against *Blastomyces* by immunodiffusion. Reporting states might have used different case classification criteria during 2019 before adopting the national case definition in 2020.

### Analysis

The analysis included confirmed coccidioidomycosis cases and confirmed or probable histoplasmosis and blastomycosis cases from all states that reported at least one of these fungal diseases in 2019. Analyzed data included case counts, case classification, sex, age, ethnicity and race, and the earliest recorded event month and type (symptom onset, diagnosis, laboratory test, or date reported to the county or state health department). States with coccidioidomycosis cases were classified based on incidence according to endemicity in certain states: high endemicity (Arizona and California), low endemicity (Nevada, New Mexico, Utah, and Washington), or not known to be endemic. These analytic groupings were chosen because Arizona and California report most cases. *Coccidioides* is also known to be present in Nevada, New Mexico, Utah, and Washington, although these states report fewer cases than Arizona and California, and cases reported from other states are typically travel associated (*35*). Analyses of histoplasmosis and blastomycosis cases were stratified by individual state. For each disease, state-specific incidence rates per 100,000 population by sex, age, and race and ethnicity categories were calculated using 2019 U.S. Census Bureau state-specific denominators for each category (*36*). The “other” race and ethnicity category was calculated using the two or more race variable from U.S. Census Bureau data. For seasonality, spring included March, April, and May; summer included June, July, and August; autumn included September, October, and November; and winter included December, January, and February. Descriptive analyses were completed in RStudio (version 4.0.3; R Foundation). This activity was reviewed by CDC and was conducted consistent with applicable federal law and CDC policy.*

## Results

### Coccidioidomycosis

**FIGURE 1:**
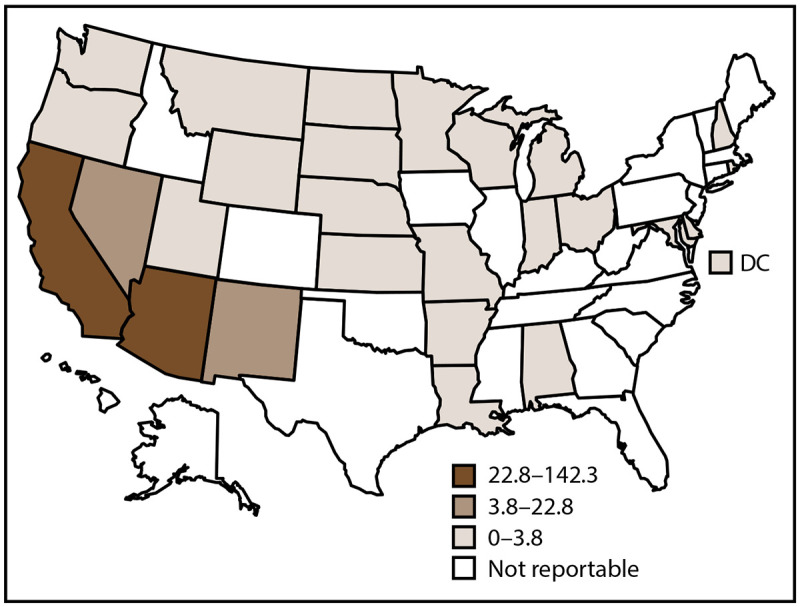
Incidence rate* of coccidioidomycosis, by state and tertiles^†,§^ — National Notifiable Diseases Surveillance System, United States, 2019 **Abbreviation:** DC = District of Columbia. * Cases per 100,000 population, calculated using state-specific denominators estimated from 2019 U.S. Census Bureau data. ^†^ Coccidioidomycosis is a reportable condition in 26 states (Alabama, Arizona, Arkansas, California, Delaware, Indiana, Kansas, Louisiana, Maryland, Michigan, Minnesota, Missouri, Montana, Nebraska, Nevada, New Hampshire, New Mexico, North Dakota, Ohio, Oregon, Rhode Island, South Dakota, Utah, Washington, Wisconsin, and Wyoming) and the District of Columbia; in Washington, coccidioidomycosis is reportable as a rare disease of public health significance. Delaware, the District of Columbia, Indiana, and Kansas reported zero cases in 2019. ^§^ In 2019, California submitted data directly to CDC.

In 2019, CDC received 20,061 coccidioidomycosis case reports from 23 of 27 states and jurisdictions where the disease is reportable (Figure 1). Delaware, the District of Columbia, Indiana, and Kansas reported zero cases. The overall incidence of coccidioidomycosis was 15.2 cases per 100,000 population. Most (19,363 [97%]) reported coccidioidomycosis cases came from Arizona and California. Arizona had the highest coccidioidomycosis incidence rate (142.3 per 100,000 population), and California had the second highest (22.8). Nevada, New Mexico, Utah, and Washington reported 2.1% (412; rate: 2.6) of coccidioidomycosis cases, and other states combined reported 1.4% (286; rate: 0.4) (Table 1).

**TABLE 1:** Number and percentage of coccidioidomycosis cases, by area and selected patient characteristics* — 23 states, 2019

Characteristic	Arizona No. (%)	California No. (%)	Nevada, New Mexico, Utah, and Washington combined^†^ No. (%)	Other states combined^§^ No. (%)	Total No. (%)
**Sex (n = 20,034**)					
Male	4,679 (45)	5,301 (59)	243 (59)	169 (59)	**10,392 (52)**
Female	5,668 (55)	3,687 (41)	168 (41)	115 (41)	**9,638 (48)**
Other	0 (0)	3 (0)	1 (<1)	0 (0)	**4 (0)**
**Age group (yrs) (n = 20,041)**					
<1	0 (0)	9 (0)	0 (0)	0 (0)	**9 (0)**
1–5	33 (<1)	51 (1)	1 (<1)	0 (0)	**85 (<1)**
6–20	650 (6)	706 (8)	25 (6)	6 (2)	**1,387 (7)**
21–40	2,111 (20)	2,596 (29)	71 (17)	28 (10)	**4,806 (24)**
41–64	3,874 (37)	3,754 (42)	172 (42)	111 (39)	**7,911 (40)**
65–80	2,925 (28)	1,489 (17)	126 (31)	117 (41)	**4,657 (23)**
>80	759 (7)	386 (4)	17 (4)	24 (8)	**1,186 (6)**
**Race and ethnicity (n = 7,846)**					
American Indian or Alaska Native, non-Hispanic	177 (8)	47 (1)	16 (6)	1 (<1)	**241 (3)**
Asian and Native Hawaiian or other Pacific Islander, non-Hispanic	72 (3)	358 (7)	11 (4)	9 (5)	**450 (6)**
Black, non-Hispanic	161 (7)	336 (6)	16 (6)	15 (8)	**528 (7)**
Hispanic or Latino (all races)	511 (23)	1,972 (38)	65 (26)	11 (6)	**2,559 (33)**
White, non-Hispanic	1,260 (56)	1,706 (33)	140 (55)	146 (77)	**3,252 (41)**
Other^¶^	51 (2)	752 (15)	5 (2)	8 (4)	**816 (10)**
**Total**	**10,359 (100)**	**9,004 (100)**	**412 (100)**	**286 (100)**	**20,061 (100)**

* Sex was missing for 27 cases, age was missing for 20 cases, and race and ethnicity was missing for 12,215 cases.

^†^ *Coccidioides* is known to be present in Nevada, New Mexico, Utah, and Washington, although these states report fewer cases than Arizona and California.

^§^ Refers to all other states where coccidioidomycosis is reportable and at least one case was reported in 2019 (Alabama, Arkansas, Delaware, Indiana, Kansas, Louisiana, Maryland, Michigan, Minnesota, Missouri, Montana, Nebraska, New Hampshire, North Dakota, Ohio, Oregon, Rhode Island, South Dakota, Wisconsin, and Wyoming).

^¶^ Calculated on basis of two or more race category.

More coccidioidomycosis cases occurred in males (10,392 [52%]; rate:15.8 per 100,000 population) than in females (9,638 [48%]; rate: 14.4). Age groups with the highest coccidioidomycosis incidence rates were 65–80 years (27.3) and >80 years (26.2) (Table 2). Coccidioidomycosis cases were most common in persons aged 65–80 years in states with low endemicity (6.4) and states in which coccidioidomycosis is not known to be endemic (1.2).

**TABLE 2:** Incidence rate* of coccidioidomycosis, by area and selected patient characteristics^†^ — 23 states, 2019

Characteristic	Arizona	California	Nevada, New Mexico, Utah, and Washington combined^§^	Other states combined^¶^	Total
**Sex (n = 20,034)**					
Male	129.4	27.0	3.0	0.2	**15.8**
Female	154.8	18.6	2.1	0.1	**14.4**
**Age group (yrs) (n = 20,041)**					
<1	0	2.0	0	0	**0.6**
1–5	7.5	2.1	0.1	0	**1.0**
6–20	45.9	9.4	0.8	0.1	**5.5**
21–40	107.7	22.6	1.6	0.2	**13.2**
41–64	186.8	31.9	3.7	0.5	**19.9**
65–80	280.6	32.6	6.4	1.2	**27.3**
>80	285.2	30.3	3.7	1.0	**26.2**
**Race and ethnicity (n = 7,846)**					
American Indian or Alaska Native, non-Hispanic	61.7	29.0	4.7	0.2	**17.4**
Asian and Native Hawaiian or other Pacific Islander, non-Hispanic	27.4	6.0	1.0	0.4	**4.6**
Black, non-Hispanic	49.4	15.1	2.4	0.2	**4.0**
Hispanic or Latino (all races)	24.6	14.1	2.2	0.3	**11.2**
White, non-Hispanic	32.0	11.8	1.4	0.3	**4.1**
Other**	33.4	67.5	1.0	0.5	**24.6**
**Total **	**142.3**	**22.8**	**2.6**	**0.4**	**15.1**

* Cases per 100,000 population. State-specific denominators from estimated 2019 U.S. Census Bureau data were used for Arizona and California incidence rates. Pooled state-specific denominators from estimated 2019 U.S. Census Bureau data were used for the respective incidence rates of the Nevada, New Mexico, Utah, and Washington combined grouping; other states combined grouping; and the total grouping. U.S. Census Bureau data were not available to calculate incidence rate for sex category “other” (three cases in California and one case in Nevada, New Mexico, Utah, and Washington combined).

^†^ Sex was missing for 27 cases, age was missing for 20 cases, and race and ethnicity was missing for 12,215 cases.

^§^ *Coccidioides* is known to be present in Nevada, New Mexico, Utah, and Washington, although these states report fewer cases than Arizona and California.

^¶^ Refers to all other states where coccidioidomycosis is reportable and at least one case was reported in 2019 (Alabama, Arkansas, Louisiana, Maryland, Michigan, Minnesota, Missouri, Montana, Nebraska, New Hampshire, North Dakota, Ohio, Oregon, Rhode Island, South Dakota, Wisconsin, and Wyoming).

** Calculated on basis of two or more races category.

Race and ethnicity data were available for 7,846 (39%) coccidioidomycosis cases (Table 1). Among those with available information, coccidioidomycosis cases were most common in non-Hispanic White (White) (3,252 [42%]) and Hispanic or Latino (Hispanic) (2,559 [33%]) persons. Non-Hispanic Black or African American persons accounted for 7% (528) of overall coccidioidomycosis cases. A substantial proportion of coccidioidomycosis cases occurred in Hispanic persons in states with high endemicity (2,483 [34%]) as well as in states with low endemicity (65 [26%]). Overall, coccidioidomycosis incidence rates by race and ethnicity were highest in non-Hispanic American Indian and Alaska Native (AI/AN) persons (17.4 per 100,000 population) and in Hispanic persons (11.2) (Table 2).

**FIGURE 2:**
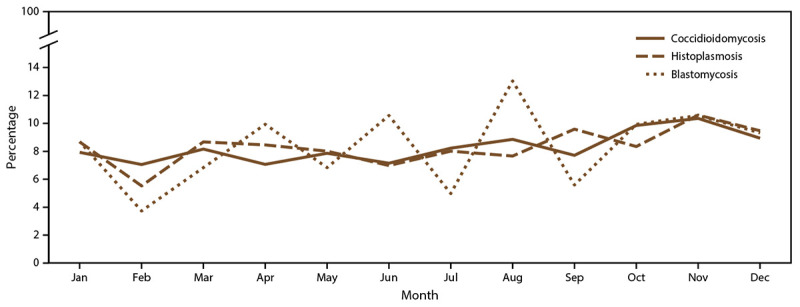
Percentage of coccidioidomycosis, histoplasmosis, and blastomycosis cases,* by month — United States, 2019 * Denominator is total cases reported in 2019 with a known earliest recorded event month for each fungal disease (coccidioidomycosis: 19,889 cases; histoplasmosis: 887 cases; and blastomycosis: 161 cases).

Earliest event date type was available for 10,964 (59%) coccidioidomycosis cases, with diagnosis date being the most common in states with high endemicity (6,093 [59%]). Event date type was not available for California, which submitted one composite episode date reflecting the earliest reported data available. Coccidioidomycosis symptom onset date was more commonly reported from states with low endemicity (316 [77%]) and states in which the disease is not known to be endemic (127 [66%]). Data on the earliest event month were submitted for 19,889 coccidioidomycosis cases (99%). Overall, coccidioidomycosis case counts were relatively consistent throughout spring (4,631 [23%]), summer (4,860 [24%]), and winter (4,797 [24%]) and slightly higher in autumn (5,601 [28%]) (Figure 2). States with low endemicity had peaks in summer (29%). In states in which coccidioidomycosis is not known to be endemic, coccidioidomycosis cases were more likely to occur in spring (31%) (Supplementary Figure 4, https://stacks.cdc.gov/view/cdc/118605).

### Histoplasmosis

**FIGURE 3:**
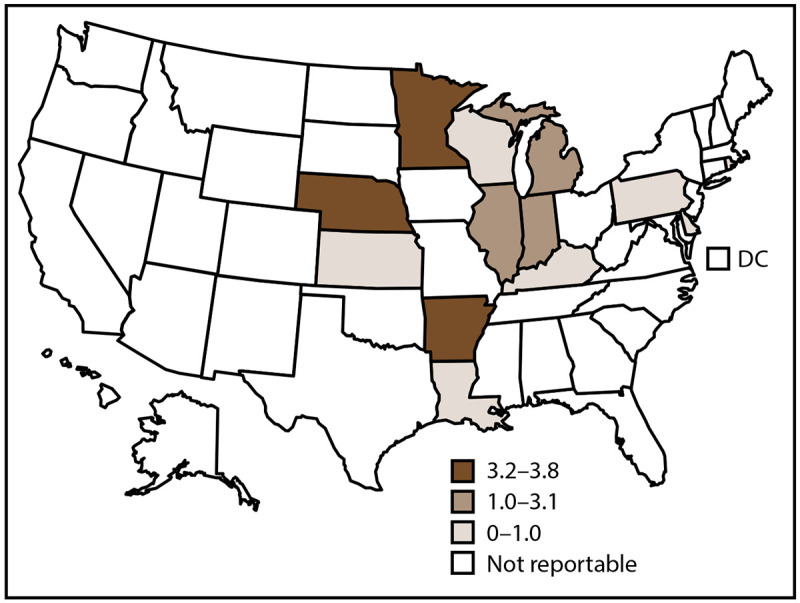
Incidence rate* of histoplasmosis, by state and tertiles^†^ — United States, 2019 **Abbreviation:** DC = District of Columbia. * Cases per 100,000 population, calculated using state-specific denominators estimated from 2019 U.S. Census Bureau data. ^†^ Histoplasmosis is a reportable condition in 13 states (Arkansas, Delaware, Illinois, Indiana, Kansas, Kentucky, Louisiana, Michigan, Minnesota, Nebraska, Pennsylvania, Rhode Island, and Wisconsin); in Rhode Island, histoplasmosis is reportable as a rare or unusual condition. Rhode Island reported zero cases in 2019. Certain state health departments submit data directly to CDC.

In 2019, CDC received 1,124 case reports of histoplasmosis from 12 states where the disease is reportable (Figure 3); Rhode Island reported zero histoplasmosis cases. The overall incidence of histoplasmosis in these states was 1.8 cases per 100,000 population. Illinois (292 [26%]; rate: 2.3), Michigan (225 [20%]; rate: 2.3), and Minnesota (214 [19%]; rate: 3.8), accounted for most histoplasmosis cases (Table 3).

**TABLE 3:** Number and percentage of histoplasmosis cases, by state, selected patient characteristics, and clinical outcomes* — 12 states,^†^ 2019

Characteristic	Arkansas No. (%)	Delaware No. (%)	Illinois No. (%)	Indiana No. (%)	Kansas No. (%)	Kentucky No. (%)	Louisiana No. (%)	Michigan No. (%)	Minnesota No. (%)	Nebraska No. (%)	Pennsylvania No. (%)	Wisconsin No. (%)	Total No. (%)
**Sex (n = 1,119)**													
Male	45 (48)	0 (0)	160 (55)	61 (57)	7 (50)	30 (65)	12 (60)	133 (59)	133 (62)	29 (43)	7 (44)	14 (58)	**631 (56)**
Female	49 (52)	0 (0)	131 (45)	46 (43)	7 (50)	16 (35)	8 (40)	92 (41)	81 (38)	39 (57)	9 (56)	10 (42)	**488 (44)**
**Age group (yrs) (n = 1,124)**													
<1	0 (0)	0 (0)	0 (0)	2 (2)	0 (0)	0 (0)	0 (0)	0 (0)	0 (0)	0 (0)	0 (0)	0 (0)	**2 (<1)**
1–5	2 (2)	0 (0)	1 (<1)	2 (2)	0 (0)	0 (0)	1 (5)	1 (<1)	2 (1)	0 (0)	0 (0)	0 (0)	**9 (1)**
6–20	15 (16)	0 (0)	34 (12)	23 (21)	0 (0)	7 (15)	2 (10)	17 (8)	22 (10)	8 (12)	0 (0)	4 (17)	**132 (12)**
21–40	32 (34)	1 (100)	79 (27)	36 (33)	1 (7)	11 (24)	9 (43)	60 (27)	51 (24)	18 (27)	2 (13)	3 (13)	**303 (27)**
41–64	25 (26)	0 (0)	119 (41)	28 (26)	9 (64)	22 (48)	7 (33)	101 (45)	83 (39)	29 (43)	8 (50)	9 (38)	**440 (39)**
65–80	20 (21)	0 (0)	49 (17)	14 (13)	3 (21)	5 (11)	2 (10)	41 (18)	50 (23)	12 (18)	5 (31)	8 (33)	**209 (19)**
>80	1 (1)	0 (0)	10 (3)	3 (3)	1 (7)	1 (2)	0 (0)	5 (2)	6 (3)	1 (2)	1 (6)	0 (0)	**29 (3)**
**Race and ethnicity (n = 859)**													
American Indian or Alaska Native, non-Hispanic	0 (0)	0 (0)	0 (0)	0 (0)	0 (0)	0 (0)	0 (0)	1 (<1)	1 (<1)	2 (3)	0 (0)	0 (0)	**4 (<1)**
Asian and Native Hawaiian or other Pacific Islander, non-Hispanic	1 (2)	0 (0)	7 (3)	2 (2)	1 (8)	0 (0)	9 (47)	0 (0)	5 (3)	0 (0)	0 (0)	0 (0)	**25 (3)**
Black, non-Hispanic	12 (21)	0 (0)	34 (17)	15 (16)	0 (0)	0 (0)	1 (5)	8 (5)	5 (3)	3 (5)	1 (14)	0 (0)	**79 (9)**
Hispanic or Latino (all races)	5 (9)	0 (0)	27 (13)	5 (5)	0 (0)	2 (8)	7 (37)	7 (4)	3 (2)	4 (6)	1 (14)	2 (9)	**63 (7)**
White, non-Hispanic	38 (68)	0 (0)	133 (66)	70 (73)	12 (92)	22 (88)	2 (11)	137 (77)	159 (92)	58 (87)	5 (71)	20 (91)	**656 (76)**
Other^§^	0 (0)	0 (0)	2 (4)	4 (7)	0 (0)	1 (2)	0 (0)	25 (45)	0 (0)	0 (0)	0 (0)	0 (0)	**32 (4)**
**Hospitalization (n = 460)**													
Yes	36 (69)	—^¶^	—	62 (63)	10 (71)	—	—	—	101 (49)	27 (42)	—	13 (54)	**249 (54)**
No	16 (31)	—	—	36 (37)	4 (29)	—	—	—	107 (51)	37 (58)	—	11 (46)	**211 (46)**
**Death (n = 415)**													
Yes	2 (8)	—	—	5 (5)	2 (14)	—	—	—	10 (5)	1 (2)	—	0 (0)	**20 (5)**
No	23 (92)	—	—	93 (95)	12 (86)	—	—	—	196 (95)	47 (98)	—	24 (100)	**395 (95)**
**Total**	**95 (100)**	**1 (100)**	**292 (100)**	**108 (100)**	**14 (100)**	**46 (100)**	**21 (100)**	**225 (100)**	**214 (100)**	**68 (100)**	**16 (100)**	**24 (100)**	**1,124 (100)**

* Sex was missing for five cases, race and ethnicity was missing for 265 cases, hospitalization status was missing for 664 cases, and death status was missing for 709 cases.

^†^ Rhode Island reported zero cases in 2019.

^§^ Calculated on basis of two or more race category.

^¶^ Not available.

More histoplasmosis cases occurred in males (631 [56%]; rate: 1.8) than in females (488 [44%]; rate: 1.3 per 100,000 population). The age groups with the highest incidence rates for histoplasmosis were 41–64 years (2.0) and 65–80 years (2.2) (Table 4).

**TABLE 4:** Incidence rate* of histoplasmosis, by state and selected patient characteristics^†^ — 12 states,^§^ 2019

Characteristic	Arkansas	Delaware	Illinois	Indiana	Kansas	Kentucky	Louisiana	Michigan	Minnesota	Nebraska	Pennsylvania	Wisconsin	Total
**Sex (n = 1,119)**													
Male	3.0	0	2.6	1.8	0.5	1.4	0.5	2.7	4.7	3.0	0.1	0.5	**1.8**
Female	3.2	0	2.0	1.4	0.5	0.7	0.3	1.8	2.9	4.0	0.1	0.3	**1.3**
**Age group (yrs) (n = 1,124)**													
<1	0	0	0	2.5	0	0	0	0	0	0	0	0	**0.2**
1–5	1.1	0	0.1	0.5	0	0	0.3	0.2	0.6	0	0	0	**0.2**
6–20	2.5	0	1.4	1.7	0	0.8	0.2	0.9	2.0	2.0	0	0.4	**1.0**
21–40	4.1	0.4	2.3	2.0	0.1	1.0	0.7	2.3	3.4	3.5	0.1	0.2	**1.6**
41–64	2.8	0	3.1	1.4	1.1	1.6	0.5	3.3	4.9	5.3	0.2	0.5	**2.0**
65–80	4.8	0	3.1	1.6	0.8	0.8	0.3	2.9	7.0	5.0	0.3	1.0	**2.2**
>80	0.9	0	2.2	1.3	0.9	0.7	0	1.4	2.9	1.4	0.2	0	**1.1**
**Race and ethnicity (n = 859)**													
American Indian or Alaska Native, non-Hispanic	0	0	0	0	0	0	0	1.8	1.7	12.3	0	0	**1.2**
Asian and Native Hawaiian or other Pacific Islander, non-Hispanic	1.7	0	1.0	1.2	1.1	0	10.8	0	1.7	0	0	0	**1.0**
Black, non-Hispanic	2.6	0	1.9	2.3	0	0	0.1	0.6	1.3	3.2	0.1	0	**0.9**
Hispanic or Latino (all races)	2.4	0	1.3	1.2	0	1.3	3.4	1.6	1.1	2.1	0.1	0.6	**1.2**
White, non-Hispanic	1.8	0	1.7	1.3	0.6	0.6	0.1	1.8	3.6	3.8	0.1	0.4	**1.3**
Other^¶^	0	0	1.0	3.1	0	1.2	0	11.0	0	0	0	0	**2.3**
**Total**	**3.2**	**0.1**	**2.3**	**1.6**	**0.5**	**1.0**	**0.5**	**2.3**	**3.8**	**3.5**	**0.1**	**0.3**	**1.6**

* Cases per 100,000 population. State-specific denominators from estimated 2019 U.S. Census Bureau data were used for Arkansas, Delaware, Illinois, Indiana, Kansas, Kentucky, Louisiana, Michigan, Minnesota, Nebraska, Pennsylvania, and Wisconsin incidence rates. Pooled state-specific denominators from estimated 2019 U.S. Census Bureau data were used to calculate the incidence rate for the total grouping.

^†^ Sex was missing for five cases, and race and ethnicity was missing for 265 cases.

^§^ Rhode Island reported zero cases in 2019.

^¶^ Calculated on basis of two or more race category.

Data on race and ethnicity were submitted for 859 (76%) histoplasmosis cases. Among those with available information, most histoplasmosis cases occurred in White persons (656 [76%]) (Table 3). Incidence rates for histoplasmosis were highest in White persons (1.3 per 100,000 population), AI/AN persons (1.2), and Hispanic persons (1.2) (Table 4).

Earliest event date type was available for 683 (61%) histoplasmosis cases, with symptom onset date the most common type (591 [87%]). Data on the earliest event month were submitted for 887 (79%) cases. Overall, histoplasmosis case frequency was relatively consistent throughout spring (223 [25%]), summer (201 [23%]), and winter (210 [24%]) and was slightly higher in autumn (253 [29%]) (Figure 2).

Hospitalization status was available for 460 (41%) histoplasmosis cases, and mortality data were submitted for 415 (37%) cases from six states (Table 3). Of these cases, 54% (249) of patients were hospitalized, and 5% (20) of patients died.

### Blastomycosis

**FIGURE 4:**
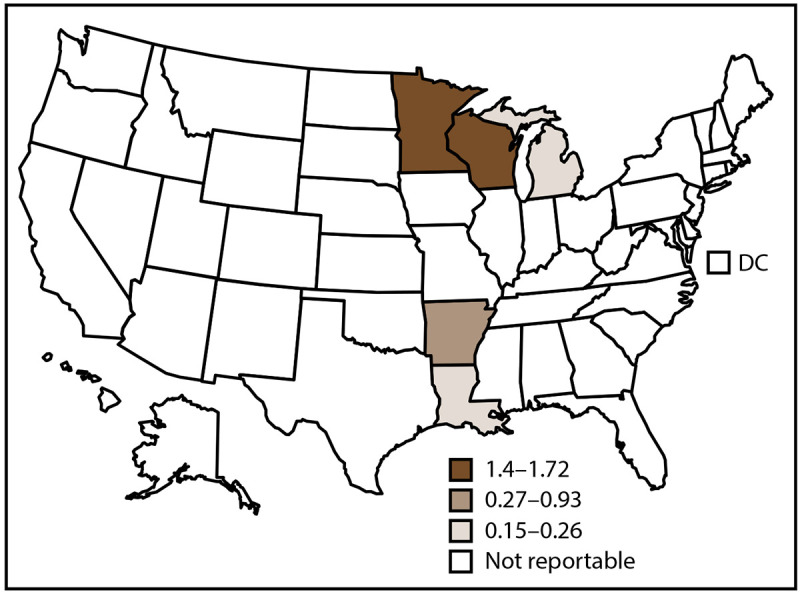
Incidence rate* of blastomycosis, by state and tertiles^†^ — United States, 2019 **Abbreviation:** DC = District of Columbia. * Cases per 100,000 population, calculated using state-specific denominators estimated from 2019 U.S. Census Bureau data. ^†^ Blastomycosis is a reportable condition in five states (Arkansas, Louisiana, Michigan, Minnesota, and Wisconsin). State health departments submit data directly to CDC.

In 2019, CDC received 240 case reports of blastomycosis from five states (Figure 4). The overall blastomycosis incidence in these states was 0.8 cases per 100,000 population. Minnesota (1.4) and Wisconsin (1.7) accounted for 75% (179) of cases (Table 5).

**TABLE 5:** Number and percentage of blastomycosis cases, by state, selected patient characteristics, and clinical outcomes* — five states, 2019

Characteristic	Arkansas No. (%)	Louisiana No. (%)	Michigan No. (%)	Minnesota No. (%)	Wisconsin No. (%)	Total No. (%)
**Sex (n = 239)**						
Male	18 (64)	7 (100)	17 (65)	56 (71)	70 (71)	**168 (70)**
Female	10 (36)	0 (0)	9 (35)	23 (29)	29 (29)	**71 (30)**
**Age group (yrs) (n = 240)**						
<1	0 (0)	0 (0)	0 (0)	0 (0)	0 (0)	**0 (0)**
1–5	1 (4)	0 (0)	0 (0)	1 (1)	0 (0)	**2 (1)**
6–20	8 (29)	0 (0)	0 (0)	6 (8)	10 (10)	**24 (10)**
21–40	6 (21)	2 (29)	4 (15)	37 (47)	30 (30)	**79 (33)**
41–64	11 (39)	2 (29)	13 (50)	22 (28)	42 (42)	**90 (38)**
65–80	2 (7)	3 (43)	8 (31)	13 (16)	15 (15)	**41 (17)**
>80	0 (0)	0 (0)	1 (4)	0 (0)	3 (3)	**4 (2)**
**Race and ethnicity (n = 208)**						
American Indian or Alaska Native, non-Hispanic	0 (0)	0 (0)	1 (5)	5 (7)	4 (5)	**10 (5)**
Asian and Native Hawaiian or other Pacific Islander, non-Hispanic	1 (5)	0 (0)	0 (0)	8 (11)	6 (7)	**15 (7)**
Black, non-Hispanic	3 (15)	2 (100)	1 (5)	7 (9)	11 (13)	**24 (12)**
Hispanic or Latino (all races)	3 (15)	0 (0)	1 (5)	4 (5)	6 (7)	**14 (7)**
White, non-Hispanic	13 (65)	0 (0)	19 (86)	52 (68)	60 (68)	**144 (69)**
Other^†^	0 (0)	0 (0)	0 (0)	0 (0)	1 (1)	**1 (1)**
**Hospitalization (n = 228)**						
Yes	14 (82)	5 (83)	16 (62)	51 (65)	61 (61)	**147 (65)**
No	3 (18)	1 (17)	10 (38)	28 (35)	39 (39)	**81 (36)**
**Death (n = 224)**						
Yes	1 (8)	2 (29)	3 (12)	5 (6)	9 (9)	**20 (9)**
No	11 (92)	5 (71)	23 (88)	74 (94)	91 (91)	**204 (91)**
**Total**	**28 (100)**	**7 (100)**	**26 (100)**	**79 (100)**	**100 (100)**	**240 (100)**

* Sex was missing for one case, race and ethnicity was missing for 32 cases, hospitalization status was missing for 12 cases, and death status was missing for 16 cases.

^†^ Calculated on basis of two or more race category.

More blastomycosis cases occurred in males (168 [70%]; rate: 1.2 per 100,000 population) than in females (71 [30%]; rate: 0.5). The age groups with the highest blastomycosis incidence rates were 21–40 years (1.3), 41–64 years (1.2), and 65–80 years (1.3) (Table 6).

**TABLE 6:** Incidence rate* of blastomycosis, by state and selected patient characteristics^†^ — five states, 2019

Characteristic	Arkansas	Louisiana	Michigan	Minnesota	Wisconsin	Total
**Sex (n = 239)**						
Male	1.2	0.3	0.4	1.9	2.4	**1.2**
Female	0.7	0	0.2	0.8	1.0	**0.5**
**Age group (yrs) (n = 240)**						
<1	0	0	0	0	0	**0**
1–5	0.5	0	0	0.3	0	**0.1**
6–20	1.4	0	0	0.6	0.9	**0.5**
21–40	0.8	0.2	0.3	2.5	2.0	**1.3**
41–64	1.2	0.2	0.8	1.3	2.3	**1.2**
65–80	0.5	0.5	1.1	1.8	1.9	**1.3**
>80	0	0	0.4	0	1.4	**0.2**
**Race and ethnicity (n = 208)**						
American Indian or Alaska Native, non-Hispanic	0	0	1.8	8.3	7.6	**4.5**
Asian and Native Hawaiian or other Pacific Islander, non-Hispanic	2.1	0	0	2.7	3.4	**1.6**
Black, non-Hispanic	0.7	0.1	0.1	1.8	3.0	**0.6**
Hispanic or Latino (all races)	1.4	0	0.2	1.5	1.7	**0.9**
White, non-Hispanic	0.6	0	0.3	1.2	1.3	**0.7**
Other^§^	0	0	0	0	1.0	**0.2**
**Total**	**0.9**	**0.2**	**0.3**	**1.4**	**1.7**	**0.8**

* Cases per 100,000 population. State-specific denominators from estimated 2019 U.S. Census Bureau data were used for Arkansas, Louisiana, Michigan, Minnesota, and Wisconsin incidence rates. Pooled state-specific denominators from estimated 2019 U.S. Census Bureau data were used to calculate the incidence rate for the total grouping.

^†^ Sex was missing for one case, race and ethnicity was missing for 32 cases, hospitalization status was missing for 12 cases, and death status was missing for 16 cases.

^§^ Calculated on basis of two or more race category.

Data on race and ethnicity were submitted for 208 (87%) blastomycosis cases. Among blastomycosis cases with race and ethnicity information, most were in White persons (144 [69%]) (Table 5). Incidence rates for blastomycosis were highest in AI/AN persons (4.5 per 100,000 population) and non-Hispanic Asian and Native Hawaiian or other Pacific Islander persons (1.6) (Table 6).

Earliest event date type was available for 33 (14%) blastomycosis cases, with symptom onset date being the most common type (19 [58%]). Data on the earliest event month were submitted for 161 (67%) blastomycosis cases. Overall, blastomycosis cases were most frequent in summer (46 [29%]) and autumn (42 [26%]) and least frequent in winter (35 [22%]) (Figure 2).

Hospitalization status was available for 228 (95%) blastomycosis cases, and mortality data were submitted for 224 (93%) cases from five states (Table 5). Of these cases, 65% (147) of patients were hospitalized, and 9% (20) of patients died.

## Discussion

This report provides an update on the epidemiology of coccidioidomycosis, histoplasmosis, and blastomycosis from 2019 surveillance data. Coccidioidomycosis, histoplasmosis, and blastomycosis are associated with substantial case numbers (21,425 cases total for all three diseases), morbidity (with approximately one half of histoplasmosis and blastomycosis patients hospitalized), and deaths (5% for histoplasmosis and 9% for blastomycosis). Continued surveillance in reporting states, expansion to other states, and collection of additional data elements would allow public health professionals to better understand year-to-year trends and changing epidemiology of coccidioidomycosis, histoplasmosis, and blastomycosis to identify populations at risk more effectively and to guide public health interventions.

The findings in this report confirm that coccidioidomycosis, histoplasmosis, and blastomycosis cause substantial illness in the United States, particularly coccidioidomycosis in terms of the number of cases reported (20,061). Although substantially fewer histoplasmosis and blastomycosis cases were reported, surveillance for these two diseases occurred in fewer states than for coccidioidomycosis. Even in states where histoplasmosis and blastomycosis are reportable, missed cases are likely because milder illnesses might be less commonly detected than mild coccidioidomycosis, in part because of the broader and less concentrated geographic distributions of histoplasmosis and blastomycosis than of coccidioidomycosis (*37*,*38*). In 2019, a total of 249 histoplasmosis and 147 blastomycosis cases resulted in hospitalization. These numbers are substantially lower than those reported in previous studies (*39*,*40*), which found that histoplasmosis and blastomycosis result in approximately 5,000 and 1,000 hospitalizations, respectively, each year in the United States according to administrative data, highlighting substantial discrepancies between sources of case reports. Although counts differed considerably, the high hospitalization and mortality rates for histoplasmosis and blastomycosis described in this report align with past surveillance reports (*8*,*14*,*39*). In-depth, state-level comparisons of surveillance and administrative hospitalization data are needed to evaluate potential gaps in reporting and accuracy of administrative coding data.

The acquisition of environmental diseases can be influenced by health disparities and is more likely to afflict vulnerable and minority populations (*41*). The data in this report confirm health disparities in the occurrence of these fungal diseases. AI/AN persons are more commonly affected by endemic mycoses, with blastomycosis incidence approximately six times as high and coccidioidomycosis incidence approximately four times as high as incidence in White persons. Asian and Native Hawaiian or other Pacific Islander persons had blastomycosis incidence approximately twice as high as White persons. Hispanic persons had coccidioidomycosis incidence rates almost three times as high as White persons. Incidence differences between other race and ethnicity categories for these three diseases were substantially smaller, although the relatively low proportion of data completeness for race and ethnicity suggests caution in interpreting these rates. Reasons for differences in reported incidences in endemic mycoses are not fully understood. The markedly higher coccidioidomycosis and blastomycosis rates for AI/AN persons might reflect in part greater population concentration in areas of higher incidence, although more specific exposures (e.g., occupational or recreational) might have a role. These disparities also might be related to chronic occupational, housing, and health care access discrimination. Disparities in insurance status or access to health care also might impede timely diagnosis and appropriate treatment (*42*,*43*). Understanding the associations of disease incidence and social vulnerabilities might help further explain the differences in reported incidences. Granularity in race and ethnicity data could identify populations at risk and help focus public health action; for example, persons of Hmong ancestry might be at higher risk for severe blastomycosis (*27*,*44*).

Coccidioidomycosis, histoplasmosis, and blastomycosis cases were more common in males, consistent with previous studies (*8*,*14*,*30*), although with substantial differences by pathogen, ranging from 52% of coccidioidomycosis infections to 70% of blastomycosis infections in males. Biologic differences and occupational exposure might explain why endemic mycoses occur more commonly in males. A recent study suggests that biologic differences might be a factor for higher rates and severity of coccidioidomycosis in males, raising the question of whether such factors also have a role in histoplasmosis and blastomycosis (*45*). The predominance of cases in males also might be related to occupational exposure, particularly for outdoor occupations traditionally held by males (e.g., construction, excavation, landscaping, hunting, or agricultural work) (*46*); expanded surveillance could help in understanding the impact of these occupations (*8*,*14*,*47*). Although risk factors for hospitalization could not be determined because of the summary format of the data, other reports have identified males to be at increased risk for hospitalization related to histoplasmosis and coccidioidomycosis (*14*,*48*). Reports of hospitalization rates by sex for blastomycosis indicate conflicting results; further research is needed in this area (*8*,*40*).

Seasonal trends exhibited slight yet meaningful variations for all three fungal diseases throughout the year. Symptom onset date is rarely reported from states where coccidioidomycosis endemicity is high, and the use of laboratory report dates with associated lags might obscure seasonal trends. State-specific differences in how earliest event date is calculated as well as the incubation periods of coccidioidomycosis (7–21 days), histoplasmosis (14–28 days), and blastomycosis (median: 45 days) also might influence observed seasonality (*37*,*38*,*49*). Differences in peak coccidioidomycosis cases occurred in Arizona and California. A dual peak occurred in summer and autumn in Arizona, whereas California had only an autumn peak, which past surveillance reports also identified (*30*). A recent report suggested that wet winters accompanied by hot summers after dry years might increase the risk for coccidioidomycosis in California (*50*). Although exposure sources could not be ascertained, the spring peak in coccidioidomycosis cases reported from states where the disease is not known to be endemic is consistent with previous reports and likely stems from travelers spending the colder months in areas where *Coccidioides* is present and later returning to their permanent residence (*9*,*30*). Cases peaked in autumn for histoplasmosis and in winter for blastomycosis; however, increases were relatively small compared with other seasons. Studies suggest no seasonal variation with histoplasmosis hospitalizations whereas blastomycosis cases are more commonly reported in autumn and winter; however, certain disease presentations like pulmonary and disseminated disease might appear more often during different seasons of the year (*39*,*51*).

Previous studies have suggested that climate change could expand the geographic range of climate-sensitive fungi, especially *Coccidioides, Histoplasma*, and *Blastomyces. Coccidioides* is now detected as far north as Washington, and a predictive modeling study suggested that by the year 2100, the niche environment for *Coccidioides* could expand to dry western states including Montana, Nebraska, North Dakota, and South Dakota, which have not previously had locally acquired infections (*4*,*11*). Recent enhanced surveillance reports described a substantial number of histoplasmosis cases in Michigan, Minnesota, and Wisconsin, north of the previously defined area, suggesting northward expansion of hospitable habitats for *Histoplasma (**14*). Dozens of blastomycosis cases have been reported in patients living outside of the disease’s previously established endemic range in states such as Kansas, Nebraska, New York, Texas, and Vermont (*52*–*54*). Surveillance for coccidioidomycosis, histoplasmosis, and blastomycosis does not capture information from all states, which limits the understanding of the changes in geographic distribution of these diseases. Expanding surveillance to additional states would help create a more accurate picture of the geographic distribution and could provide standardized case data for further analysis with other data (e.g., weather, environment, or soil moisture) to monitor effects of climate change on disease risk.

Public health surveillance for these fungal diseases through NNDSS does not capture information related to exposures, occupation, disease presentation, antifungal treatment, hospitalization, or death. Efforts to improve public health surveillance by systematically collecting certain data elements would improve understanding of nationwide disease and treatment patterns, emerging populations at risk, and exposure sources (*55*). Certain state and local health departments are collecting such information through their local surveillance or cluster investigation efforts; specific message-mapping guides for coccidioidomycosis, histoplasmosis, and blastomycosis would allow additional valuable variables to be gathered through NNDSS.

## Limitations

The findings in this report are subject to at least four limitations. First, the case counts are likely an underrepresentation of the actual case counts of coccidioidomycosis, histoplasmosis, and blastomycosis in the United States because of misdiagnosis and underreporting. Histoplasmosis and blastomycosis also are only reported by a limited number of states. Second, only summary-level data were available for most histoplasmosis cases and all blastomycosis cases, which prevented analyses of factors associated with hospitalizations and deaths. Third, multiple variables had incomplete data (e.g., race, ethnicity, or event date). For most states, outcome data were either not available or were frequently recorded as “unknown” for histoplasmosis. Finally, this report includes only 1 year of data; therefore, trends over time could not be assessed.

## Future Directions

National surveillance of coccidioidomycosis, histoplasmosis, and blastomycosis can be improved and expanded to include more states and data elements. Transitioning histoplasmosis and blastomycosis to nationally notifiable status would help with more comprehensive reporting. States where coccidioidomycosis, histoplasmosis, and blastomycosis are endemic but are not reporting nationally might consider making these diseases reportable in their state and submitting their case data to NNDSS. Robust data provided through NNDSS on exposures, treatment, and clinical characteristics could improve public health and clinical interventions for both primary and secondary prevention.

Further research about the effect of climate change on geographic spread, exposure prevention, antifungal treatment outcomes, and best practices for improving clinician and public awareness about these diseases could help reduce illness and save lives (*56*). Evidence-based clinical diagnostic algorithms could be a helpful resource for faster and more accurate diagnosis of coccidioidomycosis, histoplasmosis, and blastomycosis by health care providers across the United States. Novel antifungal treatments and vaccines would be important tools for the health care industry and policymakers to consider for the prevention of coccidioidomycosis, histoplasmosis, and blastomycosis.

## Conclusion

This report summarizes national surveillance data for coccidioidomycosis, histoplasmosis, and blastomycosis in 2019. States with the highest case numbers were Arizona and California for coccidioidomycosis; Illinois, Michigan, and Minnesota for histoplasmosis; and Minnesota and Wisconsin for blastomycosis. Compared with coccidioidomycosis, which is a nationally notifiable condition, histoplasmosis and blastomycosis are reportable in limited states. High mortality and hospitalization rates for histoplasmosis and blastomycosis suggest underreporting of mild and severe disease while highlighting their severe clinical outcomes. Health care professionals should consider these three mycoses when evaluating patients with community-acquired pneumonia or other acute lower respiratory illness who were involved in high-risk activities and live in, work in, or have traveled to areas where these fungi are known to be endemic and which might be expanding due to climate change. Improved and expanded surveillance would allow a more robust public health response to these diseases.

## Footnotes

* See e.g., 45 C.F.R. part 46.102(l)(2), 21 C.F.R. part 56; 42 U.S.C. ^§^241(d); 5 U.S.C. ^§^552a; and 44 U.S.C. ^§^3501 et seq.
